# Smoking and multiple sclerosis susceptibility

**DOI:** 10.1007/s10654-013-9853-4

**Published:** 2013-10-22

**Authors:** Anna Karin Hedström, Jan Hillert, Tomas Olsson, Lars Alfredsson

**Affiliations:** 1 Institute of Environmental Medicine, Karolinska Institutet, Stockholm, Sweden; 2 Department of Clinical Neurosciences, Karolinska Institutet, Stockholm, Sweden; 3 Centre for Occupational and Environmental Medicine, Stockholm County Council, Stockholm, Sweden

**Keywords:** Multiple sclerosis, Smoking, Case–control study

## Abstract

Smoking is one of the most established risk factors for multiple sclerosis (MS). The aim of this study was to investigate how age at smoking debut, duration, intensity and cumulative dose of smoking, and smoking cessation influence the association between smoking and MS risk. In two Swedish population-based case–control studies (7,883 cases, 9,264 controls), subjects with different smoking habits were compared regarding MS risk, by calculating odds ratios with 95 % confidence intervals. We observed a clear dose response association between cumulative dose of smoking and MS risk (*p* value for trend <10^−35^). Both duration and intensity of smoking contributed independently to the increased risk of MS. However, the detrimental effect of smoking abates a decade after smoking cessation regardless of the cumulative dose of smoking. Age at smoking debut did not affect the association between smoking and MS. Smoking increases the risk of MS in a dose response manner. However, in contrary to several other risk factors for MS that seem to affect the risk only if the exposure takes place during a specific period in life, smoking affects MS risk regardless of age at exposure, and the detrimental effect slowly abates after smoking cessation.

**Electronic supplementary material:**

The online version of this article (doi:10.1007/s10654-013-9853-4) contains supplementary material, which is available to authorized users.

## Introduction

A vast number of published investigations have assessed the link between smoking and multiple sclerosis (MS) susceptibility. Almost all have detected a significant detrimental effect [1]. A meta-analysis of published data concerning smoking and MS risk was recently updated (risk ratio 1.48, 95 % CI 1.35–1.63, *p* < 10^−15^) [1]. However, the effect of age at smoking debut, duration, intensity and cumulative dose of smoking require further clarification. In this report based on two large population-based case–control studies, we investigated aspects of the association between smoking and MS risk that have previously been investigated only to a limited extent.

## Methods

### Study design

The first study (EIMS; Epidemiological Investigation of Multiple Sclerosis) was designed as a population-based case–control study using incident cases of MS, with a study group comprising the population aged 16–70 years in geographically defined areas of Sweden. Cases were recruited via hospital-based neurology units as well as privately run neurology units in Sweden. All university hospitals participated in the study, and in total, 40 study centers reported cases of MS to the study. All cases were examined and diagnosed by a neurologist located at the unit in which the case was entered. Cases that did not fulfill the McDonalds criteria [2] at the time of this report were excluded (116 cases with clinically isolated syndrome). For each potential case, two controls were randomly selected from the national population register, taking into consideration the case’s sex, age (5-year age group) and residential area (county). If information could not be obtained from the control selected, then another control was chosen using the same principles. If a third control agreed to participate before the originally chosen controls had answered the questionnaire, all controls were included in the study. Completed questionnaires were obtained from 1,798 cases and 3,907 controls, the response proportion being 91 % for the case group and 69 % for the controls. The study period for this report was April 2005–March 2012.

In the second study (GEMS; Genes and Environment in Multiple Sclerosis), prevalent cases fulfilling the McDonald criteria were identified from the Swedish National MS-registry [3]. For each case, a control was randomly selected from the national population register matched for age, gender, and residential area at the time of the disease onset. With a response rate of 82 % for the cases and 66 % for the controls, the study comprised 6,085 cases with MS, distinct from those in EIMS, and 5,357 matched controls recruited between November 2009 and November 2011. Ethical approval for both EIMS and GEMS were obtained from the relevant ethics committees. The general structure of both studies have been reported previously [4, 5].

### Data collection and definition of smoking

In both studies, information on exposures and other circumstances was collected using a standardized questionnaire containing questions about demographic and reproductive factors, heredity, previous health, body weight and height, lifestyle factors, occupational exposures and socioeconomic circumstances. Information on smoking was obtained by asking about current and previous smoking including duration of smoking, average number of cigarettes smoked per day, and type of cigarettes. Information on exposure to passive smoking was obtained by asking if the subjects had been daily exposed to environmental tobacco smoke at home or at work (supplementary table 1). The questions on smoking habits and exposure to passive smoking were identical in both questionnaires.

For each case, the time at the initial appearance of symptoms indicative of MS was used as an estimate of the disease onset, and the year in which this occurred was defined as the index year. Tobacco smoking was considered prior to the index year in the cases and during the same period of time in the corresponding controls. Subjects who had smoked during the index year were defined as current smokers, those who had stopped smoking prior to the index year were defined as ex-smokers, and people who had never smoked before or during the index year were defined as never-smokers.

In order to analyze whether age at smoking debut affects the association between MS and smoking, we categorized the smokers into groups based on when they started and stopped smoking (age). We further categorized the smokers into groups based on the amount of cigarettes smoked (pack-years) prior to index. One pack year is defined as 20 cigarettes smoked per day for 1 year. Those who had smoked more than 10 pack years prior to index were defined as heavy smokers. Subjects who reported they had been exposed to passive smoking before or during the index year were defined as exposed to passive smoking.

### Statistical analysis

Using logistic regression, the occurrence of MS in subjects who had started and stopped smoking in different life periods was compared with that in never-smokers by calculating odds ratios with 95 % confidence intervals. Trend test for a dose response relationship regarding cumulative dose of smoking and risk of MS was performed by using a continuous variable for cumulative dose of smoking, expressed as pack-years, in a logistic regression model. In order to determine whether intensity or duration of smoking contributes most to the risk of MS, we separately examined the components comprising pack-years.

We performed matched analyses based on all available case–control sets, as well as unmatched analyses of the data based on all available cases and controls. In the matched analyses, we lost a significant number of cases and controls (2 % of the cases and 13 % of the controls in EIMS, and 42 % of the cases and 34 % of the controls in GEMS). In the matched design, each case in GEMS had one matched control (3,541 cases and 3,541 controls) whereas each case in EIMS on average had 1.92 matched controls (1,762 cases and 3,389 controls). However, the results were similar to those in the unmatched analyses and all reported results that were statistically significant in the unmatched analysis were significant in the matched analysis as well. Therefore, only the results from the unmatched analyses are presented in this report since these were in close agreement with those from the matched analyses but had a somewhat higher degree of precision.

All analyses were adjusted for age, gender, residential area (according to study design), ancestry, passive smoking, and study. In the analysis, age was categorized into the following 8 strata: 16–19, 20–24, 25–29, 30–34, 35–39, 40–45, 45–49, and 50–70 years of age. Assessment of ancestry was based on whether the subject was born in Sweden or not, and whether either of the subject’s parents had immigrated to Sweden. A subject who was born in Sweden, whose parents had not immigrated, was classified as Swedish. Passive smoking was dichotomized into those who had and had not been exposed to environmental tobacco smoke before the index year.

Other potential factors taken into consideration were heredity (having a first or second degree relative with MS or not), educational level (university degree or not), snuff use (yes/no), adolescent body mass index (≤27 versus >27 kg/m^2^), sun exposure habits during the last 5 years (high/low), and a history of infectious mononucleosis (yes/no), but these factors had minor influence on the results of the study and were not adjusted for in the final analyses. All analyses were conducted using statistical analysis system (SAS) version 9.

## Results

Our analyses of smoking and MS risk based on EIMS and GEMS included altogether 7,883 cases and 9,264 controls matched for age, gender and residential area. All cases fulfilled the McDonald criteria. The mean age at MS onset was 34 years in EIMS and 33 years in GEMS, and the mean duration from disease onset to inclusion in the study was 4.6 years in EIMS and 17.0 years in GEMS. Characteristics of cases and controls in EIMS and GEMS are presented in Table 1.

**Table 1:** Selected characteristics of cases and controls

	Ever-smokers		Never-smokers	
	Cases	Controls	Cases	Controls
Total				
Women (n, %)	3,116 (71)	3,212 (75)	2,598 (74)	3,602 (73)
Men (n, %)	1,258 (29)	1,088 (25)	911 (26)	1,362 (27)
Scandinavian origin (n, %)	3,899 (90)	3,779 (88)	3,115 (89)	4,300 (87)
Large conurbations (n, %)	1,974 (45)	1,725 (40)	1,395 (40)	1,780 (36)
Passive smoking (n, %)	1,766 (40)	1,900 (44)	1,403 (40)	1,733 (35)
Snuff users (n, %)	668 (15)	760 (17)	223 (6)	397 (8)
Obesity (BMI > 27 kg/m^2^)	265 (6)	182 (4)	206 (6)	2,011 (4)
Infectious mononucleosis (n, %)	541 (12)	332 (8)	476 (14)	414 (8)
Age at disease onset (SD)	34.6 (10.2)		32.0 (10.7)	
Total (n, %)	4,374 (100)	4,300 (100)	3,509 (100)	4,964 (100)
EIMS				
Women (n, %)	686 (71)	1,315 (74)	615 (74)	1,511 (71)
Men (n, %)	282 (29)	452 (26)	215 (26)	629 (29)
Scandinavian origin (n, %)	816 (84)	1,491 (84)	727 (88)	1,753 (82)
Large conurbations (n, %)	276 (29)	476 (27)	201 (24)	510 (24)
Passive smoking (n, %)	503 (52)	889 (50)	312 (38)	710 (33)
Snuff users (n, %)	217 (22)	400 (23)	81 (10)	236 (11)
Obesity (BMI > 27 kg/m^2^)	91 (9)	81 (5)	70 (8)	111 (5)
Infectious mononucleosis (n, %)	139 (14)	172 (10)	162 (20)	209 (10)
Age at disease onset (SD)	35.5 (10.4)		33.0 (10.5)	
Total (n, %)	968 (100)	1,767 (100)	830 (100)	2,140 (100)
GEMS				
Women (n, %)	2,430 (71)	1,897 (75)	1,983 (74)	2,091 (74)
Men (n, %)	976 (29)	636 (25)	696 (26)	733 (26)
Scandinavian origin (n, %)	3,083 (91)	2,288 (91)	2,388 (90)	2,547 (91)
Large conurbations (n, %)	1,698 (50)	1,249 (49)	1,194 (45)	1,270 (45)
Passive smoking (n, %)	1,263 (37)	1,011 (40)	1,091 (47)	1,023 (41)
Snuff users (n, %)	451 (13)	360 (14)	142 (5)	161 (6)
Obesity (BMI > 27 kg/m^2^)	171 (5)	101 (4)	136 (5)	100 (4)
Infectious mononucleosis (n, %)	402 (12)	160 (6)	314 (12)	205 (7)
Age at disease onset (SD)	34.4 (10.2)		31.6 (10.7)	
Total (n, %)	3,406 (100)	2,533 (100)	2,679 (100)	2,824 (100)

Stockholm, Göteborg, or Malmö

Compared with subjects classified as never smokers, ever smokers had an increased risk of developing MS (OR 1.5 (95 % CI 1.4–1.6, *p* < 10^−36^). When EIMS and GEMS were analyzed separately, the OR for MS among ever smokers was 1.6 (95 % CI 1.4–1.8, *p* < 10^−10^) in EIMS, and 1.5 (95 % CI 1.4–1.7, *p* < 2^−21^) in GEMS. When separate analyses were made for women and men, female ever-smokers had an OR for MS of 1.4 (95 % CI 1.3–1.5, *p* < 10^−19^) compared with female never-smokers. The corresponding result for men was 1.7 (95 % CI 1.6–2.0, *p* < 10^−19^).

Age at start of regular smoking did not seem to affect the risk of developing MS neither among current nor among past smokers (Table 2). A subanalysis was performed comprising subjects who had started smoking after the age of 25 [this category was not included in Table 2 since the numbers were too small to allow classification of past smokers (70 cases and 77 controls)]. Compared with never-smokers, the OR of developing MS among those who started smoking after the age of 25 was 1.6 (95 % CI 1.2–2.1) among current smokers and 1.4 (1.0–1.9) among past smokers.

**Table 2:**
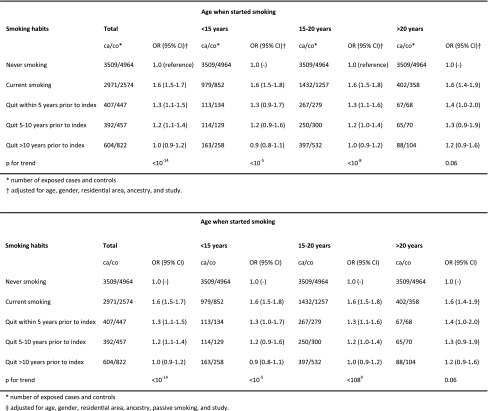
OR with 95 % CI of developing MS for current and past smokers compared with never-smokers, by age at starting smoking

The increased risk of MS associated with current and past smoking was more pronounced among heavy smokers (Table 3). Trend test for a dose response relationship between smoking and MS risk, using a continuous variable for cumulative dose of smoking, rendered a *p* value of <10^−35^. There were significant trends for increasing MS risk both with increasing duration (*p* for trend <10^−31^) and with increasing intensity of smoking (*p* for trend <10^−30^). When duration and intensity of smoking were mutually adjusted for one another in the same multivariate model, both factors contributed independently to the risk of MS (*p* for trend <10^−26^ and 10^−7^ respectively) (Table 4). However, the increased risk of MS associated with smoking abates a decade after smoking cessation regardless of the timing of smoking (Table 2) and regardless of the cumulative dose of smoking (Table 3). All analyses were adjusted for All analyses were adjusted for age, gender, residential area (according to study design), ancestry, passive smoking, and study (when appropriate). A number of other factors associated with MS risk were also considered with regard to their potential confounding effect. These were heredity, snuff use, adolescent BMI, sun exposure habits and a history of infectious mononucleosis, but the confounding effect from these turned out to be negligible.

**Table 3:**
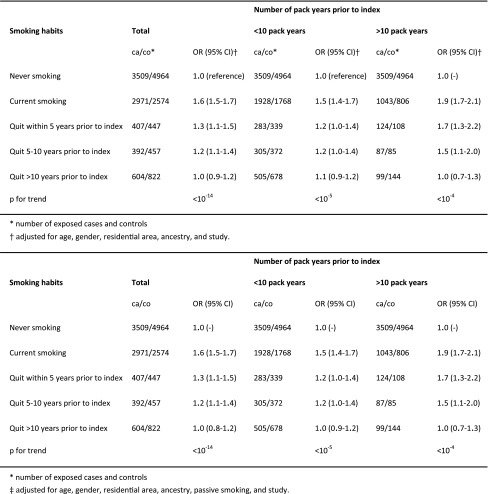
OR with 95 % CI of developing MS for current and past smokers compared with never-smokers, by cumulative dose of smoking

**Table 4:**
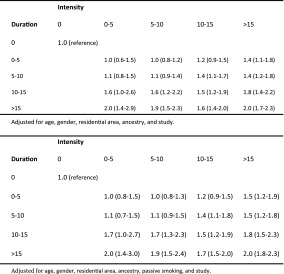
OR with 95 % CI of developing MS for smokers compared with never-smokers, by duration (years) and intensity of smoking (number of cigarettes smoked daily)

## Discussion

We observed a clear dose–response relationship between cumulative dose of smoking and MS risk. Both duration and intensity of smoking, but not age at debut of smoking, seemed to affect the association between smoking and increased MS risk. Even smoking less than 5 cigarettes per day for many years implied a two-fold increased risk of developing MS. However, the detrimental effect of smoking abates a decade after smoking cessation regardless of cumulative dose of smoking and age at exposure.

Both family studies and migration studies suggest that the influence of environmental factors contribute to MS at different age periods [1]. Established environmental risk indicators include low exposure to ultraviolet radiation [6] and vitamin D deficiency [7], Epstein–Barr virus [8] and smoking. The effects of vitamin D deficiency are likely to have an impact early in life, whereas Epstein–Barr virus infection probably influences risk during adolescence or early adulthood [1]. High body mass index during adolescence or early adulthood has been associated with increased risk of developing MS later in life [9, 10]. Similarly, exposure to shift work during this age period have been associated with increased MS risk [5]. Some aspects of adolescence thus seem to be critical regarding the impact of several environmental factors on MS risk. Smoking, on the contrary, affects MS risk regardless of age at exposure, and the detrimental effect abates a decade after smoking cessation.

Both studies were designed as population-based case–control studies, in which information regarding smoking habits was collected retrospectively. In EIMS, we primarily included incident cases of MS who had received the diagnosis within the past year whereas GEMS is based on prevalent cases. Hence the probability of recall errors is higher in GEMS than in EIMS. In both studies, we took great effort to obtain the information on smoking in an identical way for the cases and the controls. The questionnaire contained a wide range of questions regarding many potential environmental risk factors and no section in the questionnaire was given prime focus. Furthermore, when EIMS and GEMS were analyzed separately, the association between smoking and MS was similar in both studies. Therefore, the potential recall bias is likely to be small in this study.

Another concern is that the recruitment of cases and controls may introduce selection bias. Some cases may have been unidentified in our study, for example those who were diagnosed in private clinics not participating in our studies. However, considering the structure of the public Swedish health care system, which provides equal free of charge access to medical services for all Swedish citizens, we believe almost all cases of MS are referred to neurological units and it is therefore not likely that the relatively few unidentified cases would cause a substantial bias in our calculations. In EIMS, the proportion of responders with regard to participation in the study was 91 % for cases and 69 % for controls and in GEMS, the proportion of responders was 82 % for cases and 66 % for controls. A potential selection bias may result from the relatively high proportion of non-responder among the controls. However, this bias is probably modest because life style habits such as prevalence of smoking and snuff use and the pattern of alcohol consumption among the controls was consistent with that expected for the general population in similar ages [11]. Furthermore, the proportion of controls who reported a high education was almost the same as the proportion of the Swedish population with a university degree in similar ages. Likewise, the distribution of socioeconomic status among controls was in line with that of the general population [11].

Cigarette smoke causes oxidative stress and proinflammatory responses in cells of the lung. Furthermore, post-translational modifications of proteins in the lungs occur as a consequence of smoking which may profoundly affect their antigenicity [12, 13]. It is thus possible that the mechanism linking smoking and MS susceptibility involves autoimmunity against proteins with post-translational modifications that are cross-reactive with CNS antigens. This hypothesis is supported by several recent findings. Smoking, but not use of moist snuff, increases MS risk, suggesting that it is not a systemic tobacco effect that alters the risk, but lung irritation [4]. Autoimmune memory cells are present and available for triggering in the lungs. In EAE studies, these cells strongly proliferate after local stimulation of the lungs and, after assuming migratory properties, reach the CNS with inflammation as a consequence [14]. Furthermore, both with regard to risk of MS and rheumatoid arthritis, interactions may exist between smoking, HLA alleles, and autoimmunity to posttranslationally modified proteins [15, 16]. This is consistent with class II allele specific recognition of particular altered self peptides in the lungs, with ensuing organ specific inflammatory disease depending on preferential peptide binding by allelic variants of class II molecules. The hypothesis of the pivotal role of the lungs in smoking-associated MS susceptibility is in line with our findings that cumulative dose of smoking increases the risk of MS in a dose response manner and that the age of the exposure is not crucial.

In conclusion, smoking affects MS risk regardless of age at smoking debut. There is a clear dose response association between cumulative dose of smoking and MS risk. Both duration and intensity of smoking seem to independently contribute to the increased risk of MS. However, the detrimental effect of smoking slowly abates after smoking cessation regardless of the cumulative dose of smoking.

## Electronic supplementary material

Below is the link to the electronic supplementary material.

Supplementary material 1 (DOC 26 kb)
