# Supplementary material for: Smoking and multiple sclerosis susceptibility
Source: Eur J Epidemiol. 2013 Oct 22;28(11):867–74. doi: 10.1007/s10654-013-9853-4 (PMC3898140; doi:10.1007/s10654-013-9853-4)
Supplement: Supplementary file 1 — Supplementary material 1 (DOC 26 kb) [file 10654_2013_9853_MOESM1_ESM.doc]

**Supplementary table 1**. Questions in EIMS and GEMS regarding smoking habits and exposure to passive smoking.

**Smoking habits**

1. If you **do not smoke**, and **have never smoked**, put a cross in the box and proceed to the section “Do you use snuff?” on page 7.

2. If you **smoke regularly**, or **have smoked regularly**, put a cross in the box.
Below, specify time period and amount.

Try to specify the average amount. Nb! The number is specified **per day**.

From age To age Number of cigarettes per day
 Number of cigars/cheroots per day Number of pipe fills per day

3. If you **smoke non-regularly**, for example at parties, or **have smoked non-regularly**, put a cross in the box. Below, specify time period and amount.

Try to specify the average amount. Nb! The number is specified **per week**.

From age To age Number of cigarettes per week
 Number of cigars/cheroots per week Number of pipe fills per week

**Exposure to passive smoking**

1. Have you ever lived with one or more persons that have smoked inside the home on a daily basis?

No Yes If “yes”, during the years:

2. At your work place, have you daily spent time in rooms in which people smoke?

No Yes If “yes”, during the years:
